# Local adaptation to precipitation in the perennial grass *Elymus elymoides*: Trade‐offs between growth and drought resistance traits

**DOI:** 10.1111/eva.13137

**Published:** 2020-10-09

**Authors:** Dana M. Blumenthal, Daniel R. LeCain, Lauren M. Porensky, Elizabeth A. Leger, Rowan Gaffney, Troy W. Ocheltree, Adrienne M. Pilmanis

**Affiliations:** ^1^ USDA‐ARS Rangeland Resources & Systems Research Unit Fort Collins CO USA; ^2^ Department of Biology University of Nevada Reno NV USA; ^3^ Department of Forest and Rangeland Stewardship Colorado State University Fort Collins CO USA; ^4^ USDI Bureau of Land Management Salt Lake City UT USA

**Keywords:** clinal variation, drought resistance, ecosystem restoration, *Elymus elymoides* (bottlebrush squirreltail), leaf osmotic potential, leaf size, local adaptation, semiarid steppe

## Abstract

Understanding local adaptation to climate is critical for managing ecosystems in the face of climate change. While there have been many provenance studies in trees, less is known about local adaptation in herbaceous species, including the perennial grasses that dominate arid and semiarid rangeland ecosystems. We used a common garden study to quantify variation in growth and drought resistance traits in 99 populations of *Elymus elymoides* from a broad geographic and climatic range in the western United States. Ecotypes from drier sites produced less biomass and smaller seeds, and had traits associated with greater drought resistance: small leaves with low osmotic potential and high integrated water use efficiency (δ^13^C). Seasonality also influenced plant traits. Plants from regions with relatively warm, wet summers had large seeds, large leaves, and low δ^13^C. Irrespective of climate, we also observed trade‐offs between biomass production and drought resistance traits. Together, these results suggest that much of the phenotypic variation among *E. elymoides* ecotypes represents local adaptation to differences in the amount and timing of water availability. In addition, ecotypes that grow rapidly may be less able to persist under dry conditions. Land managers may be able to use this variation to improve restoration success by seeding ecotypes with multiple drought resistance traits in areas with lower precipitation. The future success of this common rangeland species will likely depend on the use of tools such as seed transfer zones to match local variation in growth and drought resistance to predicted climatic conditions.

## 1 INTRODUCTION

As the climate warms, more frequent and severe droughts are predicted for much of western North America (Seager & Vecchi, 2010; Swain & Hayhoe, 2015). Many plant species will be forced to migrate and/or evolve in order to persist (Corlett & Westcott, 2013; Etterson & Shaw, 2001; Franks, 2011), and land managers will face difficult decisions about whether and how to intervene in those processes (Rice & Emery, 2003; Richardson et al., 2009). In particular, ecosystem restoration becomes challenging in the context of a changing environment (Balachowski & Volaire, 2018; Harris et al., 2006; Jones & Monaco, 2009). Managers need to identify genotypes that can be successful under both current and future climatic conditions (Doherty et al., 2017; Rice & Emery, 2003; St Clair et al., 2013). Effective management will therefore require an understanding of the patterns and mechanisms of local adaptation (McKay et al., 2005). Specifically, it is important to know how populations within a species vary in key characteristics, such as productivity and drought resistance traits, and whether that variation can be predicted from climate of origin. This information can then be used to inform seed selection for restoration, even as local climates change.

Long‐term provenance studies with trees have provided evidence for clinal variation in both drought resistance and growth rate (Dutkowski & Potts, 2012; Isaac‐Renton et al., 2018; Montwe et al., 2016). There is also strong evidence for clinal variation within herbaceous species (Baughman et al., 2019), including variation in both growth rate and leaf economic traits, which regulate potential growth rates (Albert et al., 2010; Butterfield & Wood, 2015; Etterson, 2004; Johnson et al., 2015; Parsons et al., 2011). Less is known about intraspecific variation in drought resistance or traits that confer drought resistance in herbaceous species, but there are examples. *Mimulus guttatus* plants from arid areas flower early and have relatively succulent, pubescent leaves (Kooyers et al., 2015). *Poa secunda* leaf width and size are lower in warmer, drier areas (Johnson et al., 2015), adaptations which can contribute to both leaf cooling and resistance to cavitation (Scoffoni et al., 2011; Wright et al., 2017; Yates et al., 2010). In *Chameaecrista fasciculata*, plants from warm, dry areas have thicker leaves, and this pattern is reinforced by present‐day selection patterns in their sites of origin (Etterson, 2004). With decreasing latitude, water use efficiency increases in *Panicum virgatum* (Aspinwall et al., 2013), while embolism resistance, summer dormancy, and drought survival increase in *Dactylis glomerata* (Bristiel et al., 2018; Volaire et al., 2018). To better understand the frequency and strength of such local adaptation to drought, we need broad‐scale tests (Johnson et al., 2015) that include many ecotypes from across wide climatic ranges.

Another knowledge gap, from both basic and applied perspectives, is how frequently plants experience trade‐offs between growth and drought resistance. Across species, traits that allow for rapid growth often lead to inefficient use of limited resources, and therefore reduced success in stressful, resource‐poor environments (Chapin 1980, Grime et al. 1997, Tilman 1990, Wright et al. 2004). Traits that confer rapid growth are also associated with those that lead to high water use and lower drought tolerance, particularly across broad taxonomic groupings (Bartlett, et al., 2012; Brodribb et al., 2007; Reich, 2014; Sack et al., 2013). However, such patterns are less consistently observed among more closely related species (Blackman et al., 2010; Craine et al., 2013; Ocheltree et al., 2016). Within species, clear trade‐offs between growth and drought resistance have been measured in trees across a climate gradient (Montwe et al., 2016). A few examples also exist within herbaceous species. For example, *Elymus glaucus* ecotypes from areas with lower summer water deficits are both more productive and less drought‐tolerant than those from areas with greater summer water deficits (Balachowski & Volaire, 2018). Similarly, productive ecotypes have been found to have low water use efficiency in *Panicum virgatum* (Aspinwall et al., 2013) and poor survival following summer drought in *Dactylis glomerata* (Bristiel et al., 2018).

If such trade‐offs between growth and drought resistance are common, they pose an important problem for restoration of arid and semiarid ecosystems. The species and populations that can grow and become established most rapidly may also be least tolerant of stress (Martínez‐Garza, Bongers, & Poorter, 2013, Ray‐Mukherjee, Jones, Adler, & Monaco, 2011). For example, cultivars are often used for restoration in arid systems and are frequently selected for use based on their ability to rapidly produce above‐ground biomass and large numbers of seeds (Lambert et al., 2011; Leger & Baughman, 2015). Such selection can carry with it less desirable characteristics such as reduced cold tolerance (Schroder & Prasse, 2013) and lower establishment success in stressful environments (Kulpa & Leger, 2013). It is unknown, however, whether selection of germplasm for high productivity also leads to lower drought resistance. To better understand relationships between climate, growth, and drought resistance, it is necessary to evaluate these traits together, in plants with diverse climatic origins.

Here, we describe a common garden study of drought resistance traits and growth in 99 ecotypes of the perennial grass *Elymus elymoides* [Raf.] Swezey (bottlebrush squirreltail), collected from a wide range of climates and geographic locations, and including a subset of commonly used cultivars. *Elymus elymoides* occurs in desert, grassland, shrub steppe, and forest across much of western North America (Clary, 1975). It is considered to be a key species in the restoration of ecosystems that have been degraded by anthropogenic disturbance, annual grass invasion, and wildfire (Jones & Monaco, 2009; Parsons et al., 2011). By focusing on this species, we hope to provide a broad case study of clinal variation in drought tolerance and productivity as well as information that can improve restoration success in the region. We asked three questions: (a) Can drought resistance traits and productivity be predicted from the home climate of an ecotype? Specifically, we consider traits related to dehydration avoidance (δ^13^C) and tolerance (leaf π_o,_ leaf dry matter content (LDMC), and leaf size) (Volaire, 2018) (b) Are there trade‐offs between growth and drought resistance traits? And (c) do cultivars differ from wild ecotypes in their productivity and drought resistance traits?

## 2 MATERIALS AND METHODS

### 2.1 Study species

**Figure 1:**
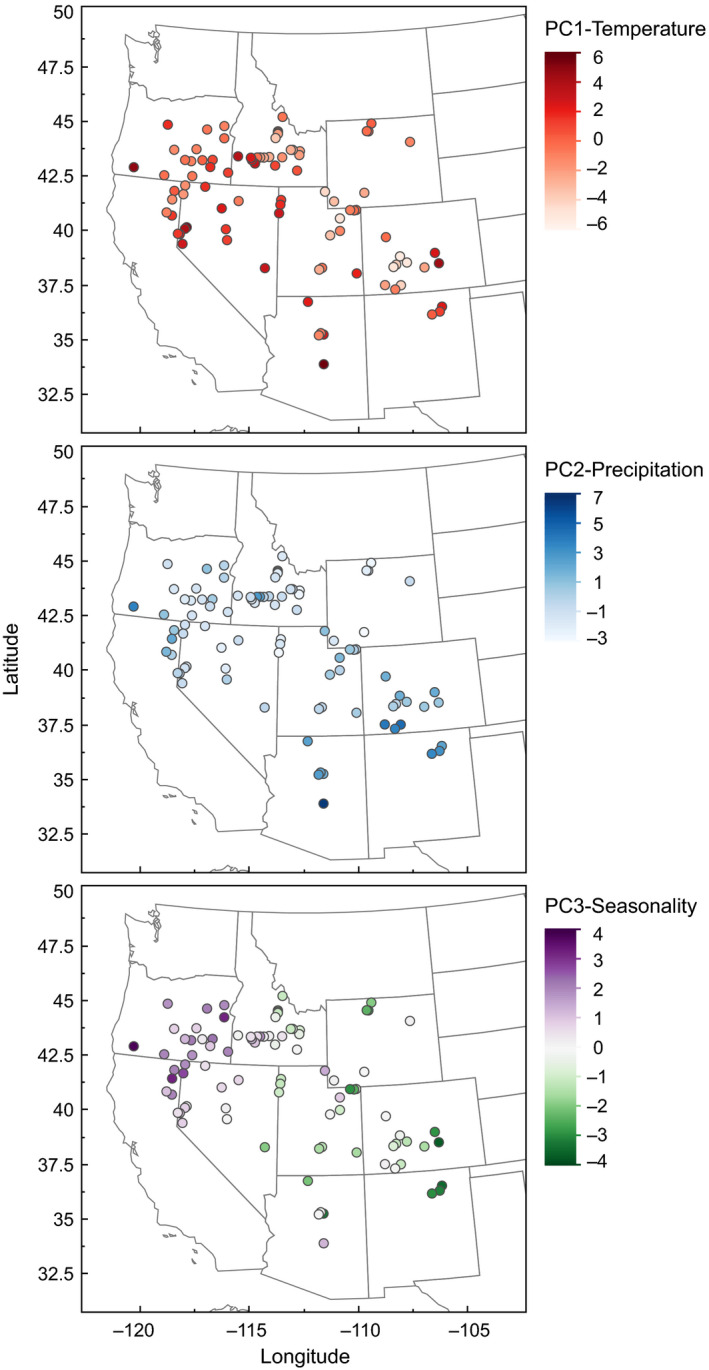
Distribution of populations across climatic gradients in the western United States. Maps depict (a) PC1, which describes mean annual and seasonal temperatures (higher values are warmer), (b) PC2, which describes mean precipitation (higher values are wetter), and (c) PC3, which describes seasonality of both precipitation and temperature; higher numbers denote warmer, wetter winters, and cooler, drier summers; lower number denote cooler, drier winters, and warmer, wetter summers

*Elymus elymoides* (bottlebrush squirreltail) is a widespread perennial grass species of the semiarid Great Basin and western Great Plains of North America. It is commonly included in restoration seed mixes due to its strong seed dispersal, rapid germination, and fire tolerance (Leger & Baughman, 2015; Parsons et al., 2011). It is also ideal for the present study due to the availability of seed from across most of its range (Figure 1) and previous work demonstrating clinal variation in traits such as phenology and productivity (Clary, 1975; Parsons, Jones, & Monaco, 2011, 2011).

Within the range of *E. elymoides*, large areas of the Intermountain Region have been dramatically altered by overgrazing, fire, and invasion of exotic species, and are now the focus of restoration efforts (Leger & Baughman, 2015). Ecosystems in the western Great Plains are more intact, but restoration following land abandonment or energy development is increasingly common (Preston & Kim, 2016). Across both regions, global climate changes are predicted to increase temperatures and precipitation variability, and therefore the frequency and intensity of drought (Kunkel *et al*. 2013; Saxon, Baker, Hargrove, Hoffman, & Zganjar, 2005; Seager & Vecchi, 2010). Consequently, understanding how *E. elymoides* populations vary with climate is likely to be important for restoration, and therefore ecosystem function, in the western United States.

### 2.2 Experimental design and seed sources

We grew plants from 99 *E. elymoides* ecotypes in a greenhouse common garden, measured drought resistance traits and growth potential, and used linear models to predict these traits as a function of home climate. We used four drought resistance traits that can help plants to tolerate or avoid drought. Leaf osmotic potential at full turgor (π_o_) is closely associated with the turgor loss point, and indicative of a plant's ability to maintain leaf turgor as water potential declines (dehydration tolerance) (Bartlett, et al., 2012; Griffin‐Nolan et al., 2019; Majekova et al., 2019). Leaf dry matter content (LDMC) is positively correlated with cell wall investment and rigidity, which can also help to maintain leaf turgor (Markesteijn et al., 2011; Onoda et al., 2017). Small leaves appear to be a common adaptation to dry conditions, contributing to both leaf cooling (dehydration avoidance) and resistance to cavitation (dehydration tolerance) (Scoffoni et al., 2011; Wright et al., 2017; Yates et al., 2010). Finally, leaf ^13^C isotope discrimination (δ^13^C) reflects rates of photosynthesis relative to stomatal conductance integrated over the lifespan of the leaf and provides a measure of integrated water use efficiency (dehydration avoidance), with less negative values indicating higher water use efficiency (WUE) (Cernusak et al., 2013). In addition to drought resistance traits, we measured total above‐ground biomass production as an estimate of growth potential.

Seed sources included (a) 75 ecotypes from the USDA‐ARS National Plant Germplasm System (NPGS), (b) 14 wild‐collected ecotypes, to improve coverage in western Nevada, and (c) 10 cultivars obtained from commercial suppliers (Table S1). Among the 75 NPGS ecotypes, 42 were wild‐collected seed (W6), and 33 were grown for seed increase under controlled conditions after collection from the wild. Hereafter, we refer to all wild‐collected ecotypes as “wild‐collected” (*n* = 56), and all ecotypes grown under controlled conditions (including both cultivars and seed increase populations) as “grown” (*n* = 43). Although the ecotypes included multiple subspecies (Parsons, et al., 2011), they are difficult to reliably separate, and we therefore focused on relationships with home climate irrespective of subspecies designation.

We grew all plants in a single greenhouse bay (USDA‐ARS, Fort Collins, CO, USA) with 28/18°C (day/night) temperatures, 18 to 40% relative humidity, 1,000 µmol/m^2^ s^‐1^ midday radiation, and a 13‐hr photoperiod. These climate conditions are similar to spring conditions near the middle of the geographic range of the collections. On March 16, 2017, we planted four replicates of each of the 99 *E. elymoides* ecotypes in separate 4 liter pots. Multiple seeds were planted in each replicate, and seedlings were thinned within 6–12 days of germination to leave a single individual. Pots contained commercial potting soil with added slow release fertilizer (“Clasicote” slow release fertilizer (16‐9‐23)). The 396 pots were fully randomized within the greenhouse. To reduce variation associated with greenhouse microenvironments, all pots were moved within the greenhouse weekly (Hardy and Blumenthal 2008). Pots were irrigated three to five days per week, to avoid water limitation.

### 2.3 Measurements and data collection

Each plant was sampled for leaf π_o_ after ~54 days of growth following methods described by Bartlett, et al. (2012). One recently fully expanded leaf was cut from the plant (between 10:00 and 14:00 hours) and quickly sealed in a small plastic bag. These were immediately frozen with dry ice and then transferred to a −80°C freezer until measurement with a VAPRO 5520 vapor pressure osmometer (Wescor, Logan UT). Osmolarity was converted to π_o_ using the following equation: π_o_ = osmolarity * −2.3958/1,000 (Griffin‐Nolan et al., 2019). At the same time, leaf dry matter content (LDMC) and leaf area were measured on one fully expanded, rehydrated leaf per plant with standard methods (Cornelissen et al. 2003).

On May 17, after ~59 days of growth and before plants began to flower, all plants were clipped at soil level, dried at 60°C for 5 days, and weighed to obtain above‐ground (dry) biomass (hereafter, biomass). This approach was designed to estimate growth potential of young plants under favorable environmental conditions, which may differ from growth responses of mature or stressed plants. Dried plant samples were then ground to a fine powder (UDY sample mill; Fort Collins, CO) and analyzed for carbon isotopes using mass spectrometry (SIRFER; University of Utah). We measured seed mass for each ecotype by weighing the caryopsis of 10 randomly chosen filled seeds from each collection used to plant the study. Seed mass was used as a covariate in analyses of plant biomass production, to account for differences in maternal provisioning. We also included seed mass as a response variable, focusing on grown ecotypes to avoid confounding of ecotype differences with and maternal effects of home climate (see statistical analysis, below).

### 2.4 Climate data

Precipitation and temperature data for the collection location of each ecotype were obtained from the daily, 2.5 arcminute PRISM AN81d database (Daly et al. 2008; www.ocs.orst.edu/prism). For seven ecotypes, we were not able to determine collection location with sufficient precision to obtain climate information. Due to the wide geographical range sampled, our collection sites varied not only in climate, but in the timing of plant growth. For example, for lower elevation sites (<1,667 m), spring green‐up occurred ~16 days earlier than higher elevation sites (>1,667 m). Consequently, to develop meaningful seasonal climate means, we used the normalized differenced vegetation index (NDVI) from the GIMMS NDVI_3g.v0_ database (Pinzon & Tucker, 2014) for each collection location to define the start of the growing season. The NDVI time series at each collection were linearly interpolated to daily values, and the delayed moving average approach (Reed et al., 1994) was used to estimate the start of the growing season for each year (1981–2013). The average start of the growing season for each collection site was used to define the start of spring, and the year was equally partitioned between spring, summer, fall, and winter. The precipitation and temperature data were aggregated to the unique phenology, based on the start of the growing season, at each collection site. Both the PRISM and GIMMS NDVI data were queried through Google Earth Engine (Gorelick et al., 2017).

### 2.5 Data analysis

We modeled plant traits as functions of climate using general linear models. Response variables were the ecotype averages of biomass, leaf area (per leaf), seed mass, leaf π_o_, δ^13^C, and LDMC. Leaf π_o_ was log_10_‐transformed to meet model assumptions. Overall variation in each response variable was described by the coefficient of variation among ecotypes. To identify a set of independent climatic predictors from our original 15 climatic variables, we used principal component analysis. Variables were standardized by subtracting the mean and dividing by the standard deviation. Associations between climate variables and PCA axes are presented in Figure S1 and Table S2. The first three axes of the PCA were then used as predictor variables in the linear models. These models included all ecotypes for which climate data were available (*n* = 92).

To understand trait variation in relation to climate in general, and to avoid attributing the same trait variation to multiple climatic variables, we focus primarily on multivariate models including all three climate predictors. These models also included seed source (wild‐collected or grown), and interactions between source and home climate (Trait~PC1 + PC2 + PC3 + Source + PC1·Source + PC2·Source + PC3·Source; Table 1). We included seed source because maternal effects are more likely to influence environment–trait relationships for wild‐collected ecotypes than for ecotypes grown in controlled environments, including both NPGS ecotypes and cultivars. Consequently, “grown” ecotypes provide a more conservative test of clinal variation in traits. Significant interactions were investigated with post hoc analyses within “wild” or “grown,” which included the first three axes of the PCA as predictor variables. In order to present actual rather than modeled (e.g., added variable plots) data, we show graphs of traits regressed against individual climate variables for relationships that are significant in multivariate models.

**Table 1:** Statistical results (*F*‐statistic, *p‐*value) from linear models predicting *E. elymoides* biomass and drought resistance from home climate (PC1‐PC3) and seed source (wild‐collected or grown)

Predictor variables	Biomass	Seed mass	d13C	Leaf π_o_	Leaf size	LDMC
Model *R* ^2^	0.33	0.60	0.23	0.11	0.44	0.24
PC1‐Temperature	0.3, 0.9	3, 0.09	2, 0.2	0.3, 0.6	0.3, 0.6	*4, 0.06*
PC2‐Precipitation	**23, <0.0001**	**70, <0.0001**	**9, 0.004**	**8, 0.005**	**52, <0.0001**	2, 0.2
PC3‐Seasonality	0.1, 0.7	**8, 0.0068**	**9, 0.003**	0.06, 0.8	**6, 0.015**	0.5, 0.5
Source	3, 0.09	**17, <0.0001**	0.07, 0.8	0.01, 0.9	0.03, 0.9	**16, 0.0001**
Temp * Source	0.02, 0.9	1, 0.3	1, 0.3	0.02, 0.9	0.1, 0.8	*4, 0.05*
Precip * Source	**11, 0.001**	0.05, 0.8	*3., 0.06*	0.3, 0.6	*3, 0.08*	1, 0.3
Season * Source	0, 1	*4, 0.07*	0.08, 0.8	0.9, 0.3	0.5, 0.5	*3, 0.08*

Note

Predictor variables represent the first three axes from principal components analysis of 15 climate variables (Figure 1, S1). Significant effects (*p* < .05) are shown in bold, and marginally significant (0.05 < *p* < .1) interactions are italicized. Numerator and denominator degrees of freedom for *F* tests were 1 and 85 (84 in the case leaf size), respectively.

Because maximum trait values may occur at intermediate values of climate variables (Wang et al., 2006), we considered quadratic as well as linear effects of climate variables. This involved, (a) testing for evidence of quadratic effects of each individual climate variable by comparing AICc values between models without (e.g., Trait ~ PC1 + Source +PC1·Source) and with (e.g., Trait ~ PC1 + PC1·PC1 + Source +PC1·Source + PC1·PC1·Source) quadratic effects (Table S3), and (b) testing for evidence of quadratic effects in multivariate models by comparing AICc values between models with linear effects of climate variables only to those with quadratic effects identified as potentially important in step 1 (Table S4).

We tested for trade‐offs between growth and drought resistance with linear models including growth variables, collection type and their interaction as predictors, and drought traits as responses (Table 2; Drought Trait ~ Biomass + Source +Biomass·Source). These analyses included all 99 ecotypes.

**Table 2:** Results from linear models (*F*‐statistic, *p‐*value) predicting drought resistance traits from biomass together with seed source

Growth	Drought Resistance			
	Leaf size	d13C	Leaf π_o_	LDMC
Model R^2^	0.42	0.04	0.11	0.10
Biomass	**34, <0.0001**	0.2, 0.6	2, 0.2	0, 1
Source	**4, 0.045**	0.8, 0.4	0.9, 0.3	**9, 0.003**
Biomass * Source	**5, 0.03**	2, 0.1	**6, 0.02**	1, 0.2

Note

Significant effects (*p* < .05) are shown in bold. Numerator and denominator degrees of freedom for *F* tests were 1 and 95 for analyses involving biomass or seed mass, and 1 and 94 for analyses involving leaf size, respectively.

**Figure 2:**
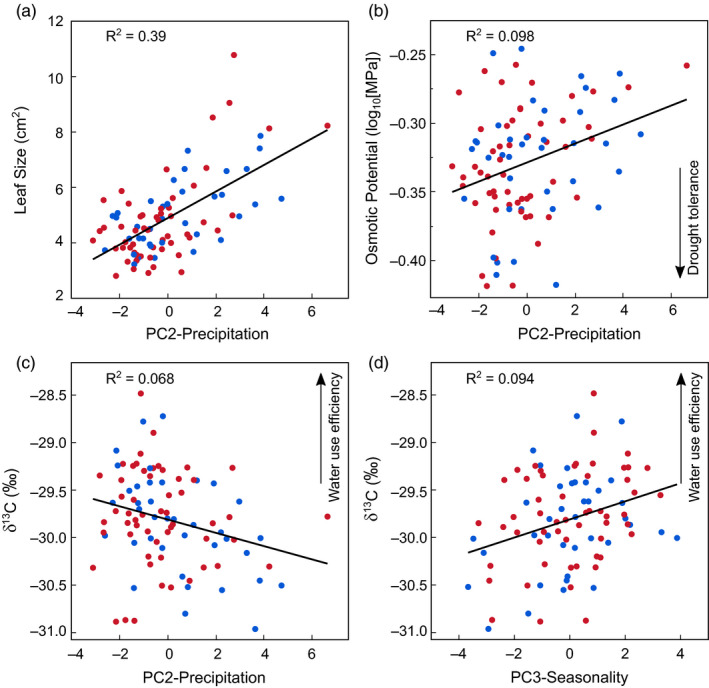
Drought resistance traits as a function of home climate, colored by seed source (red = wild‐collected; blue = grown). Plants from areas of with lower precipitation (lower values of PC2) (a) had smaller leaves, (b) had more drought‐tolerant leaves (lower π_o_), and (c) were more water use efficient (less 13C discrimination). (d) Plants from more Mediterranean climates (higher values of PC3) were also more water use efficient. Single lines indicate that interactions between climate variables and seed source were not significant

**Figure 3:**
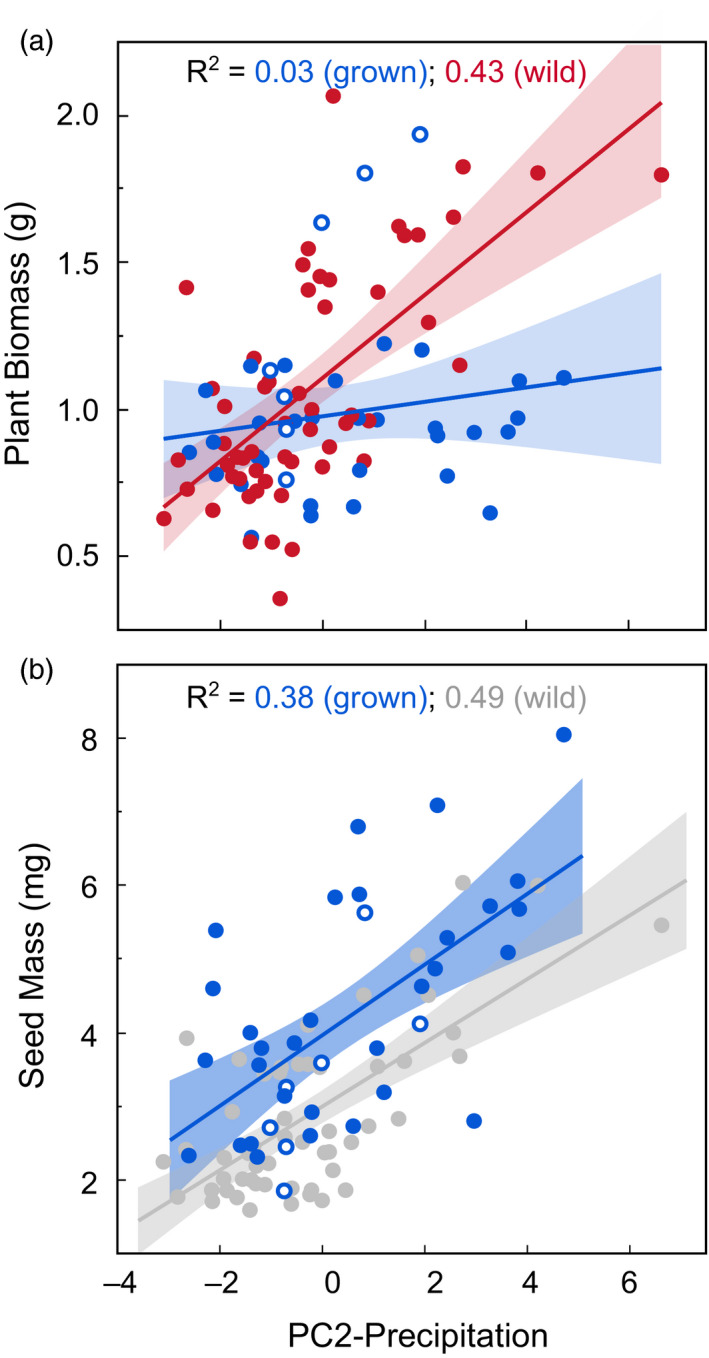
Plant biomass and seed size as a function of precipitation and seed source (red = wild‐collected; blue = grown; gray indicates a relationship that should be interpreted with caution as seed mass was measured on seeds produced in the wild rather than the common greenhouse environment). Plants from areas with higher annual and seasonal precipitation (higher values of PC2) attained more biomass. In (a), the relationship between Precipitation (PC2) and Biomass was weaker for grown than wild‐collected ecotypes (interaction *p* =0 .001). For seed mass of grown ecotypes (b), plants from areas with higher precipitation also produced larger seeds

**Figure 4:**
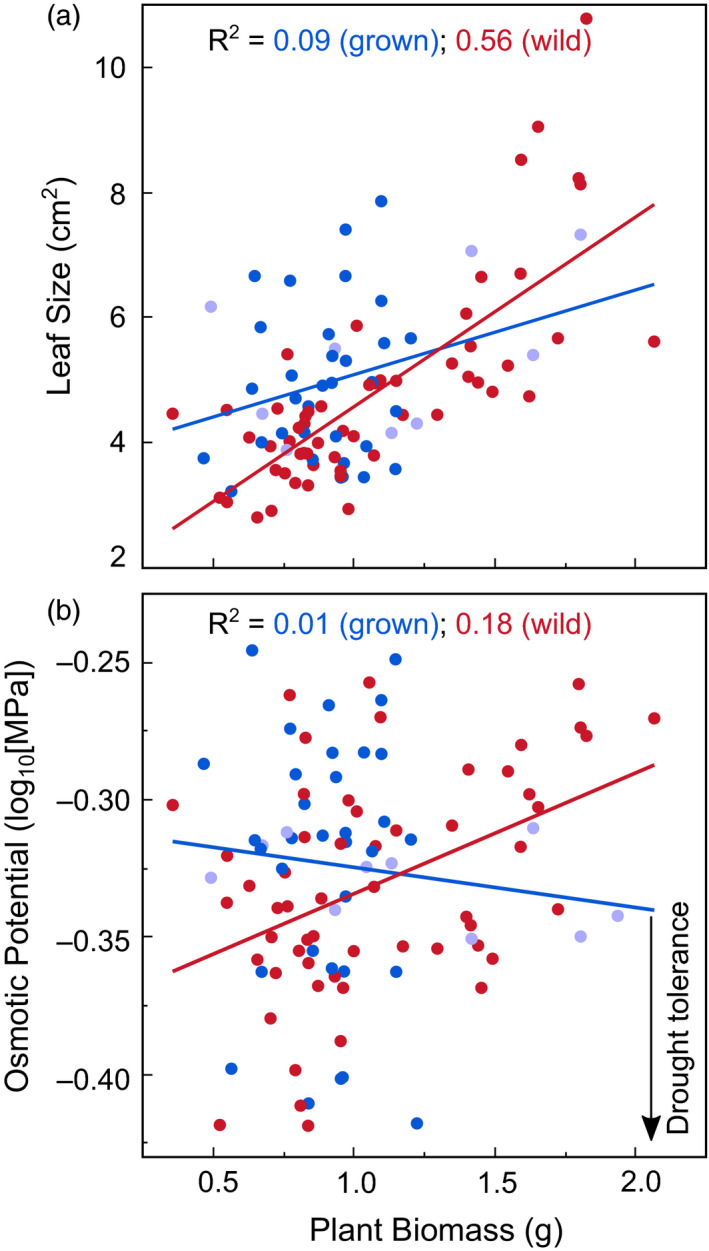
Associations between biomass and drought resistance. Larger plants had larger leaves with lower dehydration resistance (less negative π_o_). Relationships between biomass and drought traits were weaker for grown (red) than wild‐collected (blue) ecotypes (interaction P‐values < 0.03). Cultivars are shown in light blue

To test whether cultivars differed in growth or drought resistance from other collection types, we used two‐tailed *t* tests to test for differences between cultivars (*n* = 10) and wild germplasm (*n* = 89). Cultivars may also differ from other grown ecotypes in their relationships with climate variables (see open blue points in Figures 2, 3, 4). We nevertheless grouped them with other grown ecotypes in most analyses for two reasons. Most cultivars in this study were selected for particular traits, but not subsequently subjected to artificial selection, and should therefore retain relationships between their home climate and traits. In addition, our design included few cultivars (10 total, 7 associated with climate) providing little statistical power to test for distinct relationships with climate or distinct trade‐offs among traits. All analyses were conducted in JMP software (version 12, SAS).

## 3 RESULTS

### 3.1 Principal components analysis of climate

Home climates varied widely among ecotypes: Mean annual temperature ranged from 3.3°C to 12.8°C (mean of 7.8°C), and mean annual precipitation ranged from 150 mm to 787 mm (mean of 334 mm). We identified three primary axes of variation that together described 82% of the climate variation (Figure 1, S1). These axes were strongly associated with particular types of climatic variables (Table S2). “PC1‐Temperature” described variation from low to high maximum and minimum temperatures. “PC2‐Precipitation” described variation from low to high precipitation. “PC3‐Seasonality” described a spectrum from sites with more continental climates (relatively cool, dry winters and warm, wet summers) to sites with more Mediterranean climates (warmer, wetter winters and cooler, drier summers). Geographically, PC1‐Temperature was most strongly associated with elevation (*R* = −0.58), PC2‐Precipitation with latitude (*R* = −0.64), and PC3‐Seasonality with longitude (*R* = −0.75) (Figure 1).

### 3.2 Climate as a predictor of growth and drought resistance

For all response variables, we observed considerable variation among ecotypes, suggesting the potential for variation in drought resistance and growth to be associated with climate (Figures 2, 3). Coefficients of variation were greater for seed mass (44), growth (35), and leaf size (30), than for leaf π_o_ (9.6), LDMC (6.4), and δ^13^C (1.7).

We found little evidence for curvilinear responses to climate variables (Tables S3, S4). In all cases, multivariate models including only linear effects of climate variables had lower AICc values than those also including quadratic effects. Furthermore, quadratic effects of PC1‐temperature and PC3‐seasonality that were significant in univariate models were not significant in models that included all three climate predictors (the exception being a quadratic response of leaf π_o_ to seasonality). Consequently, we focus on linear responses of *E. elymoides* traits to climate (Table 1).

Leaf size, δ^13^C, and π_o_ all varied significantly with climate. Ecotypes from drier sites (low values of PC2‐Precipitation) had smaller leaves, higher WUE (less negative leaf δ^13^C), and leaves that were more resistant to dehydration (lower π_o_) (Table 1, Figure 2). A marginally significant interaction suggested that the decline in WUE with precipitation was true for grown (*R^2^* = 0.25) but not wild‐collected (*R^2^* = 0) seed (Figure S2). Ecotypes from more Mediterranean climates (high values of PC3) typical of western portions of the *E. elymoides* range also had somewhat smaller leaves (*R^2^* = 0.03) and higher WUE (Figure 2) than those from more continental climates further to the east. LDMC did not vary with climate, but was higher in grown than wild‐collected ecotypes.

Biomass and seed mass increased with home climate precipitation (Table 2, Figure 3). For biomass, this relationship was stronger among wild‐collected than grown ecotypes, potentially due in part to maternal effects mediated by seed provisioning (Figure S3). Adding seed mass to the model to control for provisioning maternal effects reduced the significance of but did not eliminate effects of precipitation (*p* = 0.01). Seed mass was also related to seasonality (*R^2^* = 0.14), being smaller in ecotypes from the more Mediterranean climates (Figure 1). For seed mass, results among wild‐collected ecotypes should be treated with caution (denoted by gray points and line in Figure 3), as seed mass was measured from seed collected in the wild and may have responded directly to differences in precipitation among collection locations.

### 3.3 Trade‐offs between growth and drought resistance

Leaf size increased with biomass production, and this increase was steeper for wild‐collected than grown ecotypes (Figure 4). Adding seed mass to the model did not influence the significance of biomass (*p < *0.0001). Among wild‐collected but not grown ecotypes, those that grew larger were also less dehydration‐resistant (higher π_o_), but this relationship was weaker (*p* = 0.1) when seed mass was added as a covariate.

### 3.4 Comparisons between cultivars and wild ecotypes

For all measured traits, cultivars were statistically indistinguishable from other ecotypes (*p* > 0.14 in all cases). Means and variances of traits for these groups are reported in Table S5.

## 4 DISCUSSION

Among *E. elymoides* ecotypes from across the western United States, we observed clinal variation in and trade‐offs among growth, leaf size, leaf π_o_, and integrated water use efficiency. Precipitation was the climatic variable most closely related to both drought resistance traits and growth. These results are in accord with previous studies showing higher growth or lower drought resistance in wetter environments (Balachowski & Volaire, 2018; Dutkowski & Potts, 2012; Etterson, 2004; Johnson et al., 2015; Montwe et al., 2016). Among studies of herbaceous species, the results also encompass an unusually wide range of ecotypes and environmental conditions, providing a broad example of how a species’ growth and drought resistance traits can vary with climate.

Climate appears to be a primary determinant of *E. elymoides* drought resistance traits, including those related to dehydration tolerance and dehydration avoidance. Ecotypes from drier areas had smaller leaves, were more resistant to dehydration (lower leaf π_o)_, and had greater WUE (higher leaf δ^13^C). Leaf size varied approximately fourfold among ecotypes, and precipitation explained 39% of that variation. This pattern in leaf size is similar to that observed for a co‐occurring perennial grass species, *Poa secunda* (Johnson et al., 2015), and also fits with associations between leaf width and aridity in other grasses (Balachowski & Volaire, 2018; Johnson et al., 2017). The strength of the relationship between leaf size and precipitation in this study, together with related patterns in other species (Baughman et al., 2019), suggests that evolution of small leaves may be a common mechanism for local adaptation to dry conditions in steppe ecosystems.

The observed increase in leaf π_o_ with precipitation suggests that local adaptation by *E. elymoides* involves physiological as well as morphological drought tolerance traits. More broadly, these results demonstrate that leaf π_o_ and precipitation can be positively associated within a species, supporting the suggestion that π_o_ is a useful trait for understanding drought tolerance (Bartlett et al., 2012). Although leaf π_o_ is closely related to climatic range among woody species (Bartlett et al., 2012), such relationships are less clear among herbaceous species (Griffin‐Nolan et al., 2019; Majekova et al., 2019). Furthermore, one other intraspecific study showed the opposite pattern, with *Bouteloua gracilis* ecotypes from more arid sites having higher and less plastic leaf π_o_ (Bushey, 2017). The intraspecific association with precipitation observed here is in accord with the idea that low leaf π_o_ helps both species and populations of herbaceous plants to tolerate dry conditions.

While we found higher WUE in ecotypes from drier sites, these patterns were not particularly strong, with precipitation explaining 7% and seasonality explaining 9% of the variation in WUE. They also differ from results of a previous study in which WUE did not differ among 12 *E. elymoides* ecotypes (Clary, 1975). In general, intraspecific variation in WUE is relatively well studied (Kooyers, 2015). For example, both *Hymenoclea salsola* shrubs and *Pinus contorta* trees from dry areas have much higher WUE than their counterparts from wet areas (Comstock & Ehleringer, 1992; Isaac‐Renton et al., 2018). In the perennial grass, *Panicum virgatum*, WUE is higher at low latitudes (Aspinwall et al., 2013). In contrast, longitudinal studies in annuals have shown that dry conditions can select for lower WUE, rapid maturation, and therefore drought escape (Franks, 2011; Kenney et al., 2014; McKay et al., 2005). Our results fit with those observed for woody species and might reflect the greater importance of drought avoidance relative to escape for perennials (Kooyers, 2015).

In contrast to drought resistance traits, biomass production and seed mass were greater among ecotypes from wetter areas. For seed mass, precipitation explained 38% of the variation among grown ecotypes. As seed of these ecotypes was produced in controlled conditions unrelated to their home climate, the correlation with precipitation may reflect genetic differences. The increase in biomass with precipitation was stronger among wild‐collected ecotypes (*R^2^* = 0.43) than among grown ecotypes (*R^2^* = 0.03), but robust to the addition of seed mass to the model, suggesting that the association between precipitation and biomass involves both maternal and genetic effects. These results contrast with those from a common garden study of 32 *E. elymoides* ecotypes, in which productivity decreased with elevation but was not affected by precipitation or temperature (Parsons et al., 2011). The wider geographic range in the current study may have provided more power for detecting precipitation effects. The positive associations we observed between biomass and precipitation also match results observed for some other herbaceous species (Balachowski & Volaire, 2018; Butterfield & Wood, 2015; Johnson et al., 2010, 2015; St Clair et al., 2013). For herbaceous species growing in dry regions, it appears to be common for growth potential to be related to local water availability (Baughman et al., 2019).

Trade‐offs between growth and drought resistance are strong at broad taxonomic scales (Brodribb et al., 2007; Reich, 2014; Sack et al., 2013), but have also been observed within species (Balachowski & Volaire, 2018; Isaac‐Renton et al., 2018; Montwe et al., 2016). In *E. elymoides*, we found that more productive ecotypes had larger individual leaves and higher leaf π_o_, traits associated with lower dehydration tolerance (Bartlett, et al., 2012; Craine et al., 2013; Scoffoni et al., 2011). The correlation between biomass and leaf π_o_ was not observed for grown ecotypes, however, and was not robust to the inclusion of seed mass in the model (*p* = 0.1), suggesting that it could represent either maternal effects or genetic variation among ecotypes. Weaker relationships among grown ecotypes could also be due to maternal effects related to the agronomic environments in which seeds were produced rather than to home climate. Previous studies of perennial grass ecotypes across latitudinal gradients have similarly found trade‐offs between growth potential and drought resistance or drought resistance traits, including leaf width, WUE, leaf π_o_, and regrowth following drought (Aspinwall et al., 2013; Balachowski & Volaire, 2018; Bristiel et al., 2018; Bushey, 2017). Together with these studies, our results for leaf size and π_o_ suggest that intraspecific growth versus drought resistance trade‐offs may involve a variety of drought resistance traits in perennial grasses.

We found cultivars to be similar to wild germplasm (grown or wild‐collected), in both their drought resistance traits and biomass production. The similarity between cultivars and wild germplasm may be explained in part by the fact that most cultivars used in this study were subjected to little artificial selection (Table S1). In contrast, released cultivars of *Pseudoroegneria spicata* and *Bromus carinatus* grew larger and produced more seed than wild ecotypes (Johnson et al., 2010; St Clair et al., 2013), while cultivars of *Bouteloua gracilis* had relatively large leaves with low specific leaf area (Butterfield & Wood, 2015). In *Plantago lanceolata* and *Lotus corniculatus*, cultivars grew larger but were less cold‐tolerant than wild ecotypes, suggesting that trade‐offs between growth and stress tolerance can be important considerations in selecting genetic material for revegetation (Schroder & Prasse, 2013).

## 5 CONCLUSIONS AND APPLICATIONS

Together, results for leaf size, δ^13^C, π_o_, and biomass suggest that much of the geographic variation in *E. elymoides* phenotypes (Parsons, et al., 2011) represents adaptation to variation in the amount and timing of water availability. Local adaptation to precipitation makes sense for a species that occupies a broad range of arid and semiarid ecosystems. It also suggests that the success of this common rangeland species in the future will depend on the maintenance of genetic variation that can lead to local adaptation over time, as climates change. For example, drought frequency and intensity are expected to increase within the current range of *E. elymoides*, particularly in southern portions of its range (Hayhoe et al., 2018), highlighting the importance of genetic variation in drought resistance.

Land managers face formidable challenges in restoring arid and semiarid ecosystems in western North America. Plant establishment has often been poor, particularly in drier areas (Arkle et al., 2014; Knutson et al., 2014; Pilliod et al., 2017). The finding that drought resistance traits vary widely and are associated with precipitation suggests that it may be possible to improve restoration success by making greater use of drought‐resistant ecotypes (Table 1, Figure 2). Such ecotypes might include combinations of drought resistance traits, including small leaves, low osmotic potential, and high water use efficiency (see Data Availability Statement, below). Leaf size may be a particularly useful trait for selecting drought‐resistant ecotypes, as it is easily measured and strongly associated with precipitation. Drought‐resistant ecotypes may also be helpful in western portions of *E. elymoides’* range, as plants from areas with more Mediterranean climates had greater water use efficiency and smaller leaves. It is important to note, however, that associations with seasonality were weaker than those with precipitation amounts. Finally, the observation of trade‐offs between growth and drought resistance suggests that productive ecotypes may not be appropriate for planting in dry areas.

Tools such as seed transfer zones that use ecotypes’ home climate to match germplasm to suitable current and future environments (Crow et al., 2018; Doherty et al., 2017; Durka et al., 2017; Shryock et al., 2018) may be critical for sustaining *E. elymoides* abundance. Our results suggest that effective climate‐based seed transfer zones would likely be derived primarily from precipitation and seasonality of precipitation and temperature. Further, trait‐based transfer zones would benefit from inclusion of traits associated with drought resistance (e.g., St Clair et al., 2013).

In sum, clinal variation in multiple traits suggests that precipitation has been central to evolution within *E. elymoides*, resulting in ecotypes with widely varying drought resistance and productivity. By making use of this variation, it may be possible to increase restoration success and long‐term persistence of this important grassland species.

## Conflict of Interest

None declared.

## AUTHOR CONTRIBUTIONS

DMB, DRL, LMP, RG, TWO, and AMP designed the study. DRL, AMP, and EAL obtained seeds. DRL conducted the experiment. DMB, DRL, LMP, and RG analyzed the data. DMB and DRL led the writing of the manuscript. All authors contributed critically to the drafts and gave final approval for publication.

## Supporting information

Supplementary Material

## Data Availability

Ecotypic variation in Elymus elymoides productivity and drought resistance traits across the western United States. https://doi.org/10.5061/dryad.931zcrjhz.
