# Supplementary material for: Local adaptation to precipitation in the perennial grass Elymus elymoides: Trade‐offs between growth and drought resistance traits
Source: Evol Appl. 2020 Oct 9;14(2):524–35. doi: 10.1111/eva.13137 (PMC7896711; doi:10.1111/eva.13137)
Supplement: Supplementary file 1 — Supplementary Material [file EVA-14-524-s001.docx]

**Supplementary Material**

**Table S1.** Collection locations and artificial selection history of the commercially available germplasm used in the study. These accessions are considered to be pre-variety germplasm from one of three categories: germplasm not evaluated for performance but for which the origin is known (Source identified class), germplasm selected for particular traits but not subject to genetic manipulation (Selected class, natural track), and germplasm subject to artificial selection within populations (Selected class, manipulated track).

| **Accession name** | **Selection type** | **Year of release** | **Traits selected for** | **State of origin** | **Additional notes** |
| --- | --- | --- | --- | --- | --- |
| Tusas | Selected, natural track | 2001 | vigor, late flowering, seed yield | NM | Composite collection |
| Toe Jam Creek | Selected, natural track | 2003 | seed mass, seedling vigor, low awn mass | NV |  |
| Fish Creek | Selected, natural track | 2003 | seedling emergence, late seed production | ID |  |
| Wapiti | Selected, natural track | 2004 | forage and seed production, vigor, leaf abundance, height | CO | Collected in 1981 |
| Pueblo | Selected, natural track | 2005 | forage and seed production, vigor, leaf abundance, height | CO | Collected in 1976 |
| Crooked River | Source identified | 2005 | -- | OR |  |
| Rattlesnake | Selected, manipulated track | 2007 | biomass, seed production, height | ID | Composite collection |
| Pleasant Valley | Selected | 2010 | high seed yield | OR |  |
| Hanford | Source identified | -- | -- | WA |  |
| Columbia Plateau | Source identified | -- | -- | WA |  |

**Table S2.** Loading matrix for the first three principal components of the PCA. Loadings represent correlations between input variables and resulting principal components. Bold text is used to highlight loadings higher than 0.4.

| **Climate variable** | **PC1** | **PC2** | **PC3** |
| --- | --- | --- | --- |
| Annual precipitation | -0.08805 | **0.94920** | 0.17294 |
| Annual maximum temperature | **0.87898** | 0.17054 | -0.12915 |
| Annual minimum temperature | **0.94702** | -0.00223 | 0.17589 |
| Spring precipitation | -0.06816 | **0.74333** | **0.43665** |
| Spring minimum temperature | **0.71181** | 0.31216 | **-0.45953** |
| Spring maximum temperature | 0.39772 | 0.40972 | **-0.78296** |
| Summer precipitation | -0.15984 | **0.64971** | **-0.48690** |
| Summer minimum temperature | **0.76926** | -0.02418 | -0.37480 |
| Summer maximum temperature | **0.70364** | -0.18395 | **-0.41756** |
| Autumn precipitation | -0.36477 | **0.80137** | -0.01797 |
| Autumn minimum temperature | **0.80111** | -0.19934 | **0.49606** |
| Autumn maximum temperature | **0.81186** | -0.23346 | **0.42879** |
| Winter precipitation | 0.08367 | **0.64906** | **0.62550** |
| Winter minimum temperature | **0.81935** | 0.13242 | **0.45293** |
| Winter maximum temperature | **0.74828** | **0.44359** | -0.05450 |

**Figure S1.** Joint plots for the first three principal components of the PCA used to identify independent axes of climatic variation with the range of *Elymus elymoides.*

­­­


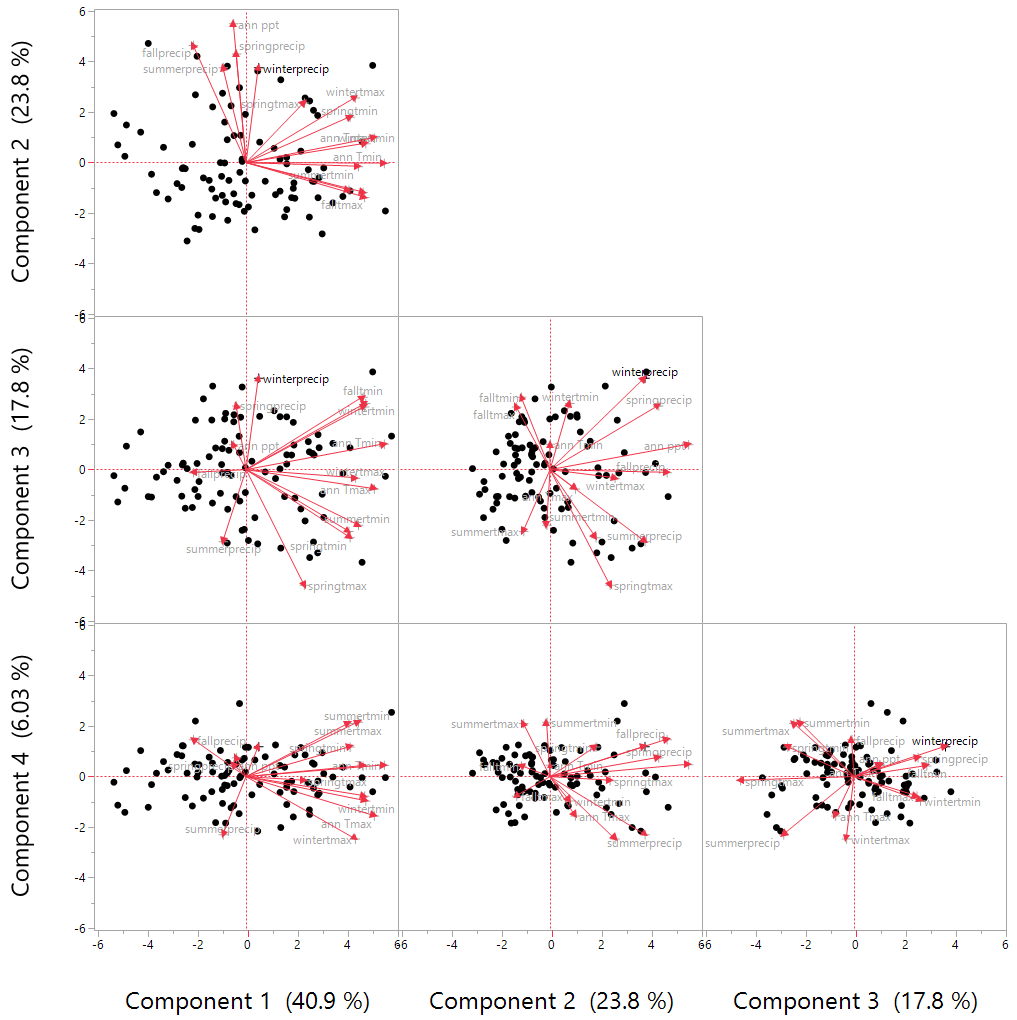

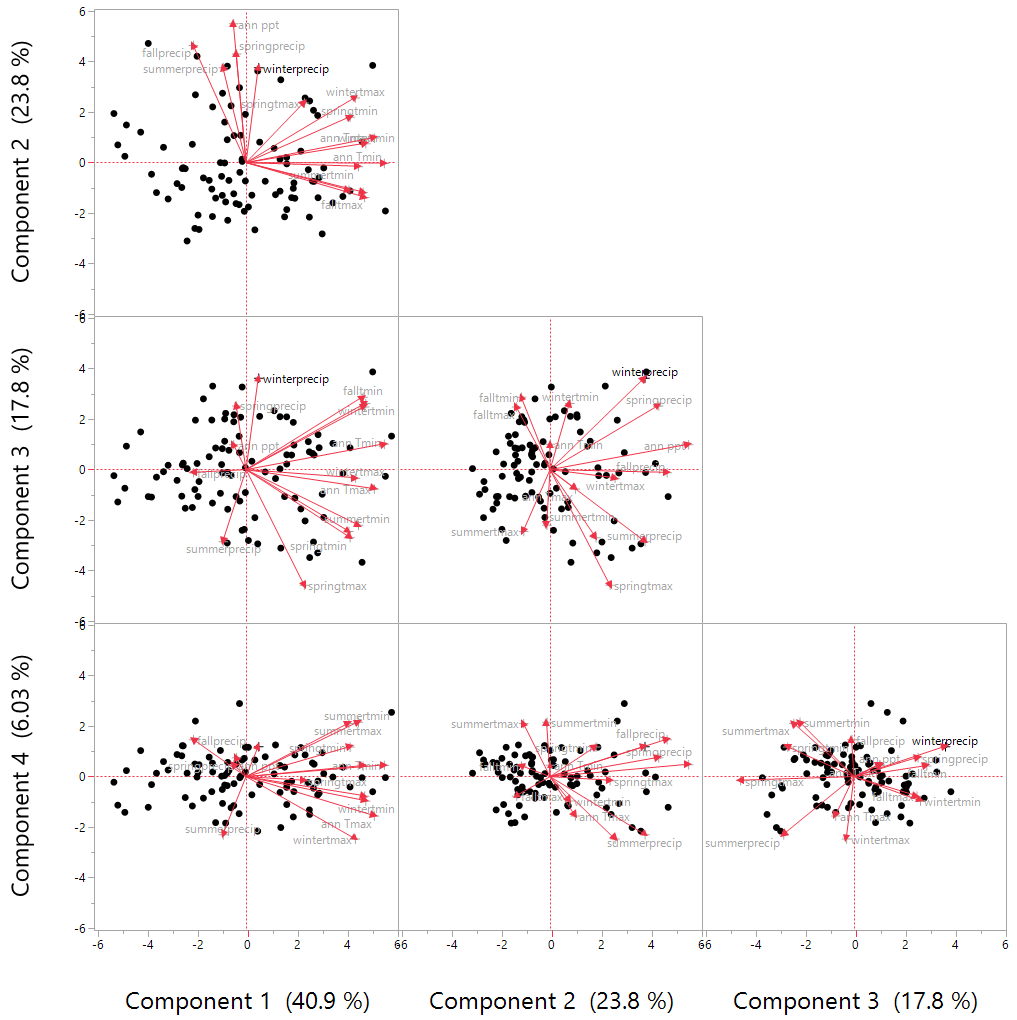


**Table S3.** Statistical results from linear models predicting *E. elymoides* biomass and drought resistance from individual climate axes and seed source (wild-collected or grown). Predictor variables represent the first three axes from principal components analysis of 15 climate variables (Figures 1, S1). Significant effects (P<0.05) are shown in bold. For each combination of predictor and response variable, models with and without quadratic effects were compared, and the more parsimonious model (lower AICc) was left unshaded. Numerator and denominator degrees of freedom for F tests were 1 and 89 (88 in the case of leaf size), for models without quadratic effects and 1 and 87 (86 in the case of leaf size) for models with quadratic effects, respectively.

| **Predictor (Statistics)** | **Biomass** | **Seed mass** | **d13C** | **Leaf π_o_** | **Leaf size** | **LDMC** |
| --- | --- | --- | --- | --- | --- | --- |
| Model R^2^ | 0.03 | 0.21 | 0.03 | 0.01 | 0.01 | 0.20 |
| AICc | 78.2 | 319.6 | 144.9 | -317.2 | 339.7 | -481.1 |
| PC1-Temperature (*F, P*) | 1, 0.3 | 1, 0.3 | 1, 0.3 | 0.2, 0.7 | 0.5, 0.5 | 3, 0.09 |
| Source (*F, P*) | 0.6, 0.5 | **18, <0.0001** | 0.1, 0.7 | 0.2, 0.7 | 0.7, 0.4 | **14, 0.0003** |
| Temp * Source (*F, P*) | 0.1, 0.7 | 0.9, 0.4 | 0.8, 0.4 | 0.05, 0.9 | 0, 1 | 4, 0.06 |
| Model R^2^ | 0.04 | 0.26 | 0.03 | 0.07 | 0.09 | 0.19 |
| AICc | 81.3 | 318.8 | 148.9 | -318.3 | 336.2 | -477.5 |
| PC1-Temperature (*F, P*) | 0.9, 0.4 | 0.9, 0.4 | 0.7, 0.4 | 0.8, 0.4 | 0.4, 0.5 | 4, 0.06 |
| Temp* Temp (*F, P*) | 2, 0.2 | **5, 0.04** | 0.05, 0.8 | **5, 0.04** | **7, 0.008** | 0.04, 0.9 |
| Source (*F, P*) | 0.6, 0.5 | 8, **0.006** | 0.04, 0.8 | 0.6, 0.5 | 0, 1 | **6, 0.02** |
| Temp * Source (*F, P*) | 0, 1 | 0.1, 0.7 | 0.7, 0.4 | 0.2, 0.7 | 0.4, 0.5 | 4, 0.06 |
| Temp * Temp * Source (*F, P*) | 0.01, 0.9 | 0.6, 0.5 | 0.6, 0.4 | 1, 0.3 | 0.5, 0.5 | 1, 0.3 |
|  |  |  |  |  |  |  |
| Model R^2^ | 0.32 | 0.54 | 0.11 | 0.10 | 0.40 | 0.14 |
| AICc | 44.3 | 269.9 | 136.4 | -326.5 | 293.5 | -476.6 |
| PC2-Precipitation (*F, P*) | **25, <0.0001** | **66, <0.0001** | **8, 0.007** | **9, 0.004** | **52, <0.0001** | 1, 0.3 |
| Source (*F, P*) | **4, 0.04** | **20, <0.0001** | 0, 1 | 0, 1 | 0.1, 0.7 | **13, 0.0006** |
| Precip * Source (*F, P*) | **12, 0.0008** | 0.2, 0.7 | **5, 0.04** | 0.3, 0.6 | 2, 0.1 | 1, 0.3 |
| Model R^2^ | 0.33 | 0.50 | 0.12 | 0.12 | 0.42 | 0.15 |
| AICc | 47.5 | 271.1 | 140.6 | -323.7 | 295.9 | -472.7 |
| PC2-Precipitation (*F, P*) | **20, <0.0001** | **27, <0.0001** | 3, 0.08 | 3, 0.09 | **25, <0.0001** | 0.9, 0.4 |
| Precip * Precip (*F, P*) | 1, 0.3 | 3, 0.08 | 0.3, 0.6 | 1, 0.33 | 0.7, 0.4 | 0.05, 0.8 |
| Source (*F, P*) | 1, 0.3 | **10, 0.002** | 0, 1 | 0.07, 0.8 | 0.03, 0.9 | 5, 0.03 |
| Precip * Source (*F, P*) | 5, **0.03** | 0.03, 0.9 | 2, 0.1 | 0.05, 0.8 | 0.6, 0.5 | 0.07, 0.8 |
| Precip * Precip * Source (*F, P*) | 0.7, 0.4 | 0.7, 0.4 | 0.01, 0.9 | 0.06, 0.8 | 0.4, 0.6 | 0.7, 0.4 |

**Table S3 (continued)**

| **Predictor/Statistics** | **Biomass** | **Seed mass** | **d13C** | **Leaf π_o_** | **Leaf size** | **LDMC** |
| --- | --- | --- | --- | --- | --- | --- |
| Model R^2^ | 0.02 | 0.26 | 0.10 | 0.02 | 0.05 | 0.14 |
| AICc | 79.2 | 313.7 | 137.9 | -318.5 | 335.9 | -476.9 |
| PC3 – Seasonality (*F, P*) | 0.3, 0.6 | 5, 0.03 | 9, 0.003 | 0.05, 0.8 | 4, 0.06 | 0.7, 0.4 |
| Source (*F, P*) | 1, 0.3 | 24, **<0.0001** | 0.4, 05 | 0.3, 0.6 | 0.5, 0.5 | **12, 0.0008** |
| Season * Source (*F, P*) | 0.07, 0.8 | 4, 0.05 | 0, 1 | 1, 0.2 | 1, 0.3 | 2, 0.1 |
| Model R^2^ | 0.06 | 0.30 | 0.12 | 0.07 | 0.10 | 0.15 |
| AICc | 79.6 | 312.9 | 139.8 | -318.9 | 335.2 | -473.2 |
| PC3 – Seasonality (*F, P*) | 0.5, 0.5 | **4, 0.045** | **8, 0.005** | 0, 1 | 3, 0.09 | 0.5, 0.5 |
| Season * Season (*F, P*) | 4, 0.06 | 3, 0.07 | 0.9, 0.4 | **5, 0.03** | 4, 0.052 | 0.8, 0.4 |
| Source (*F, P*) | 0.1, 0.7 | **10, 0.002** | 0.05, 0.8 | 0.4, 0.5 | 0, 1 | **7, 0.009** |
| Season * Source (*F, P*) | 0.1, 0.7 | 4, 0.06 | 0.01, 0.9 | 2, 0.2 | 0.9, 0.4 | 2, 0.1 |
| Season * Season * Source (*F, P*) | 0.8, 0.4 | 1, 0.3 | 1, 0.3 | 0.2, 0.7 | 0.5, 0.5 | 0, 1 |

**Table S4.** Statistical results from multivariate linear models including quadratic factors for potentially quadratic relationships identified in Table S2. Models predict *E. elymoides* biomass and drought resistance from climate axes and seed source (wild-collected or grown). Predictor variables represent the first three axes from principal components analysis of 15 climate variables (Figures 1, S1). Significant effects (P<0.05) are shown in bold. AICc values are shown for both models with linear effects of climate variables only (see Table 1 for model results), and for models with quadratic effects. For each combination of predictor and response variable, the more parsimonious model (lower AICc) was left unshaded. Numerator and denominator degrees of freedom for F tests were 1 and 85 (84 in the case of leaf size) for models without quadratic effects and 1 and 81 (80 in the case of leaf size) for models with quadratic effects, respectively.

| **Predictor (Statistics)** | **Seed mass** | **Leaf π_o_** | **Leaf size** |
| --- | --- | --- | --- |
| AICc (models with only linear effects, Table 1) | 265 | -318 | 296 |
| AICc (models with quadratic effects, below) | 269 | -317 | 301 |
| PC1-Temperature (*F, P*) | 4, 0.05 | 3, 0.1 | 0.3, 0.6 |
| PC2-Precipitation (*F, P*) | **48, <0.0001** | 2, 0.2 | **36, <0.0001** |
| PC3 – Seasonality (*F, P*) | **7, 0.009** | 0, 1 | **6, 0.02** |
| Temp* Temp (*F, P*) | 0.06, 0.8 | 3, 0.1 | 2, 0.2 |
| Season * Season (*F, P*) | 0.5, 0.5 | **5, 0.03** | 0, 1 |
| Source (*F, P*) | 09, 0.3 | 0.5, 0.5 | 2, 0.2 |
| Temp * Source (*F, P*) | 3, 0.1 | 0.07, 0.8 | 0.09, 0.8 |
| Precip * Source (*F, P*) | 0.7, 0.4 | 0.1, 0.7 | **5, 0.03** |
| Season * Source | 2, 0.1 | 1, 0.3 | 0.4, 0.5 |
| Temp * Temp * Source (*F, P*) | 1, 0.3 | 2, 0.1 | 2, 0.1 |
| Season * Season * Source (*F, P*) | 3, 0.07 | 0.07, 0.8 | 0.5, 0.5 |

|  |  |  |  |
| --- | --- | --- | --- |

**Figure S2**. Marginally significant interactions (0.05 < *P* < 0.1) between climate and seed source. See Table 1 for statistical details.


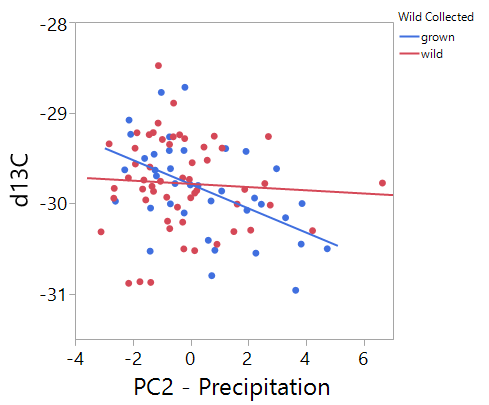

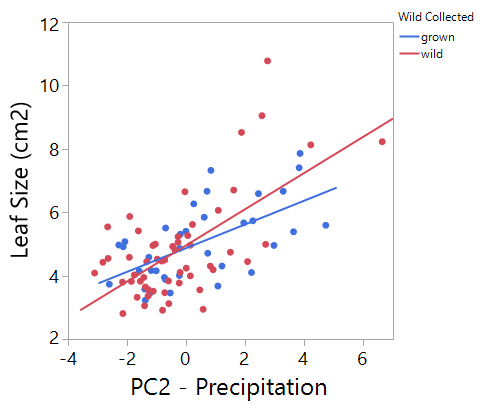

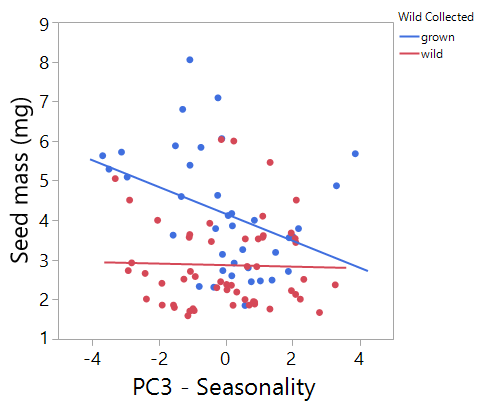

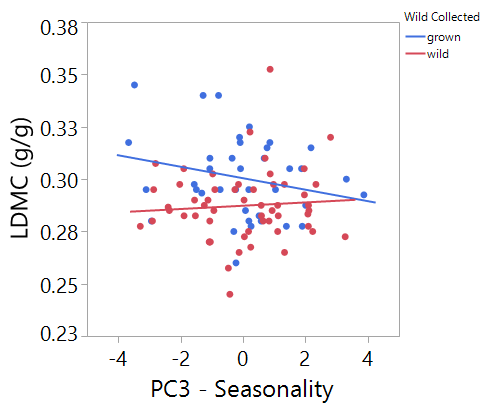


R^2^ = 0.25 (grown); 0.00 (wild)

R^2^ = 0.06 (grown); 0.01 (wild)

R^2^ = 0.37 (grown); 0.41 (wild)

R^2^ = 0.1 (grown); 0.00 (wild)

**Figure S3.** A pattern of greater biomass production in ecotypes grown from larger seeds was observed in wild-collected (*R^2^*=0.32) but not grown (*R^2^*=0.01) ecotypes. This suggested the potential for maternal effects related to offspring provisioning. To address this possibility, we investigated significant interactions involving seed source, by conducting post-hoc regressions of wild-collected ecotypes both with and without seed mass as a covariate.


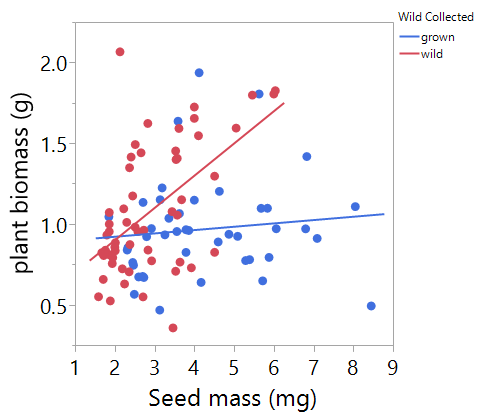


**Table S5.** Means and variances of growth and drought resistance traits for cultivars and wild ecotypes.

|  | Cultivar | | Wild | |
| --- | --- | --- | --- | --- |
|  | Mean | Variance | Mean | Variance |
| Biomass (g) | 1.18 | 0.246 | 1.01 | 0.119 |
| Seed mass (mg) | 4.16 | 4.59 | 3.40 | 2.078 |
| d13C | -29.9 | 0.398 | -29.8 | 0.249 |
| Leaf π_o_ (MPa) | -2.134 | 0.00552 | -2.14 | 0.0462 |
| Leaf size (cm^2^) | 5.33 | 1.73 | 4.85 | 2.15 |
| LDMC (g/g) | 0.294 | 0.000214 | 0.292 | 0.000372 |
